# Consumption and Vaccination

**Published:** 1899-08

**Authors:** 


### CONSUMPTION AND VACCINATION.

Says *Health* (London) anent the Tuberculosis Congress:

"It may be a satisfaction to English people to know that they suffer less from phthisis than do either the French or Germans, but this is no reason for delaying the precautions that are essential to suppress the malady altogether, or to refrain from using the means for the alleviation of the pain and misery already existing."

In Germany and France no one escapes vaccinations; in England a very large number escape it; hence less consumption. The way to stop consumption and cancer is to cease implanting them; or, in other words, vaccinating. Among the white races this superstitious rite of vaccinating is a potent cause of making the human ground ready for tuberculosis, cancer, and scrofula in various forms, while among the darker races it spreads leprosy as wind does a prairie fire.—*Homœopathic Envoy.*

